# High cholera vaccination coverage following emergency campaign in Haiti: Results from a cluster survey in three rural Communes in the South Department, 2017

**DOI:** 10.1371/journal.pntd.0007967

**Published:** 2020-01-31

**Authors:** Ashley Sharp, Alexandre Blake, Jérôme Backx, Isabella Panunzi, Robert Barrais, Fabienne Nackers, Francisco Luquero, Yves Gaston Deslouches, Sandra Cohuet

**Affiliations:** 1 Field Epidemiology Training Programme, Public Health England, London, United Kingdom; 2 Epicentre, Paris, France; 3 Operational Centre Brussels, Médecins Sans Frontières, Brussels, Belgium; 4 Ministère de la Santé Publique et de la Population, Port-au-Prince, Haiti; Yale University Yale School of Public Health, UNITED STATES

## Abstract

Oral cholera vaccine (OCV) has increasingly been used as an outbreak control measure, but vaccine shortages limit its application. A two-dose OCV campaign targeting residents aged over 1 year was launched in three rural Communes of Southern Haiti during an outbreak following Hurricane Matthew in October 2016. Door-to-door and fixed-site strategies were employed and mobile teams delivered vaccines to hard-to-reach communities. This was the first campaign to use the recently pre-qualified OCV, Euvichol. The study objective was to estimate post-campaign vaccination coverage in order to evaluate the campaign and guide future outbreak control strategies.

We conducted a cluster survey with sampling based on random GPS points. We identified clusters of five households and included all members eligible for vaccination. Local residents collected data through face-to-face interviews. Coverage was estimated, accounting for the clustered sampling, and 95% confidence intervals calculated.

435 clusters, 2,100 households and 9,086 people were included (99% response rate). Across the three communes respectively, coverage by recall was: 80.7% (95% CI:76.8–84.1), 82.6% (78.1–86.4), and 82.3% (79.0–85.2) for two doses and 94.2% (90.8–96.4), 91.8% (87–94.9), and 93.8% (90.8–95.9) for at least one dose. Coverage varied by less than 9% across age groups and was similar among males and females. Participants obtained vaccines from door-to-door vaccinators (53%) and fixed sites (47%). Most participants heard about the campaign through community ‘criers’ (58%).

Despite hard-to-reach communities, high coverage was achieved in all areas through combining different vaccine delivery strategies and extensive community mobilisation. Emergency OCV campaigns are a viable option for outbreak control and where possible multiple strategies should be used in combination. Euvichol will help alleviate the OCV shortage but effectiveness studies in outbreaks should be done.

## Author summary

After Hurricane Matthew hit Southern Haiti on October 4, 2016, there was an outbreak of Cholera. The Government launched a campaign to vaccinate residents using an oral vaccine, which has been proven to protect people against the disease. MSF supported the campaign in three rural areas, offering the vaccine in local clinics and going from door to door. We didn’t know how many people were living there at the time so we couldn’t say for sure if we had vaccinated enough people. To find out how many people were vaccinated we did a survey, choosing households at random and asking them if and where they received the vaccine. This showed that on average around 90% of people were vaccinated, which is a very high proportion. We can take encouragement from this that mass vaccination campaigns like this can work well, even in rural settings. Our survey showed that about half of people got their vaccine from a clinic and the other half from door-to-door vaccinators, so it’s probably important to use both approaches. Most people heard about the campaign through members of the local community called ‘criers’. This shows how important it is to engage with the local community during a vaccination campaign.

## Introduction

Cholera remains a significant problem globally, with 42 countries reporting a total of 172,454 cases, including 1304 deaths, in 2015 and periodic epidemics. There are three WHO pre-qualified oral cholera vaccines (OCVs) available: Dukoral, Shanchol and the most recent addition Euvichol [1], which was prequalified in 2015. All three vaccines use two-dose regimes. To mitigate shortages, a global stockpile of OCVs was created in 2013 for use in emergencies, with 2,242,800 doses shipped in 2015. There is growing international experience of using mass OCV as an outbreak control measure. Previous campaigns in Haiti achieved high uptake [2–4] and demonstrated effectiveness [5, 6].

In response to the increased incidence of cholera observed in the aftermath of Hurricane Matthew on October 4, 2016, a two-dose OCV campaign was conducted by the Ministry of Health Public Health and Population (MSPP), targeting residents aged over 1 year in 16 Communes in the Departments of Sud and Grande Anse. This was the first campaign to use the recently pre-qualified OCV, Euvichol. Médecins Sans Frontières (MSF) supported the vaccination campaign in three Communes of the Sud Department: Chardonnières, Côteaux, and Port-à-Piment. They delivered the first dose in all three Communes in November and December 2016 (about 4 Weeks after the hurricane at Chardonnières and Port-à-Piment and 8 weeks after at Côteaux) and provided logistical support to the MSSP for the second dose campaign in May 2017 (seven months after the hurricane). Door-to-door and fixed-site strategies were employed for both doses and mobile teams delivered vaccines to hard-to-reach communities, sometimes reachable only by foot. Vaccination coverage estimates using administrative data (based on the number of doses used divided by historical population denominators) suggested that the coverage was 61.5% in Chardonnières, 62.7% in Côteaux and 63.1% In Port-à-Piment for the first dose, however there were concerns about the reliability of the denominator given the likelihood of population movements following the hurricane. Hurricane Matthew left about 1.4 million people in need of humanitarian aid and led to significant population displacement [7, 8]. A reliable population-based assessment of the campaign performance was still lacking.

The objective of this study was to estimate the post-campaign vaccination coverage and acceptability in the communes of Chardonnières, Côteaux, and Port-à-Piment in order to evaluate the campaign, inform control measures and guide future outbreak control strategies.

## Methods

### Sampling and study population

We employed a cluster survey design using random GPS points [9–11]. The study area included the three communes of Port-à-Piment, Côteaux and Chardonnieres. The study population included all individuals eligible for vaccination (those aged over one year) who were living in the selected households during the month of the first or second dose campaign. The sample size calculation was done separately for each commune based on the narrowest age band of 1–4 years. It was set to achieve 10% precision at 95% confidence, assuming 70% coverage, a design effect of three, and 10% non-participation. This gave a sample size target for each commune of 290 children. Based on the average number of children aged 0–4 years per household in the Demographic and Health Survey 2012 [12] we required 725 households. A one-stage cluster sampling design was used with a cluster defined as the group of five households closest to each GPS point randomly drawn in georeferenced polygons of inhabited areas, meaning 145 clusters were required per Commune. Only GPS points falling on a roof or within 10 meters of a roofed structure were kept.

### Data collection

The data were collected during face-to-face interviews by trained local investigators using a standardized questionnaire in Creole or French. They collected information on vaccination status, socio-demographic status and reasons for non-vaccination. Vaccination history was based on self-report and checked against vaccination cards. Participants were shown pictures of the administration of the vaccine and the vaccination card to aid recall. When a member of the household was absent, the head of the household or the responsible adult answered the questions on their behalf and showed their vaccination cards when possible. If the household was empty or there was no adult present, a second visit was organized during the same day. If no adult was present during the second visit, the next closest household to the GPS point was selected. If five households could not be identified the GPS point was discarded and a reserve point was used. Data were entered directly onto electronic tablets using KoBo Collect. Data collection lasted from 16 June 2017 to 1 July 2017.

### Statistical analysis

For each Commune, we calculated overall and dose-specific vaccination coverage and 95% confidence intervals (95% CI). The variation of the vaccination coverage with age by sex was estimated by logistic regression using cubic splines and the 95% CI envelopes were estimated by bootstrap. Every calculation took into account the sampling method and included a finite population correction. The geographical distribution of the vaccination coverage was assessed using a general additive model, doing a binomial regression weighted by the household size. The vaccination coverage at the household level was the dependent variable, and the location of the household was the independent variable included as a smoothing spline term. We plotted the vaccination coverage alongside the standard error as an indicator of the uncertainty in the estimates. Data analysis was performed on R 3.3.4 (The R Foundation for Statistical Computing).

### Ethical considerations

This survey was conducted as part of the public health response to the cholera outbreak, in order to assess coverage and inform control measures. A formal agreement was obtained from the Ministry of Public Health for the implementation of all the components of this survey. Approval from an ethical review committee was not required. Verbal informed consent was received from participants before starting the questionnaire and documented directly on the digital form. All data were collated and analysed anonymously and no identifiable information was collected other than household coordinates.

## Results

### Characteristics of participants

The majority of GPS points represented households suitable for inclusion with just 14/435 (3%) needing to be replaced. Eight households (one in Port-à-Piment, five in Côteaux, and one in Chardonnières) did not consent to participate in the survey. Among the three Communes of Chardonnières, Côteaux and Port-à-Piment, the number of households recruited was 688, 709 and 703 respectively (total 2100), and the number of individuals recruited was 3081, 3109 and 2896 (total 9086). In the northern zone of Chardonnières house density was so low there that no cluster was selected. The age-sex distribution of the study cohort closely resembled the national population estimates [12].

### Vaccination coverage

**Fig 1:**
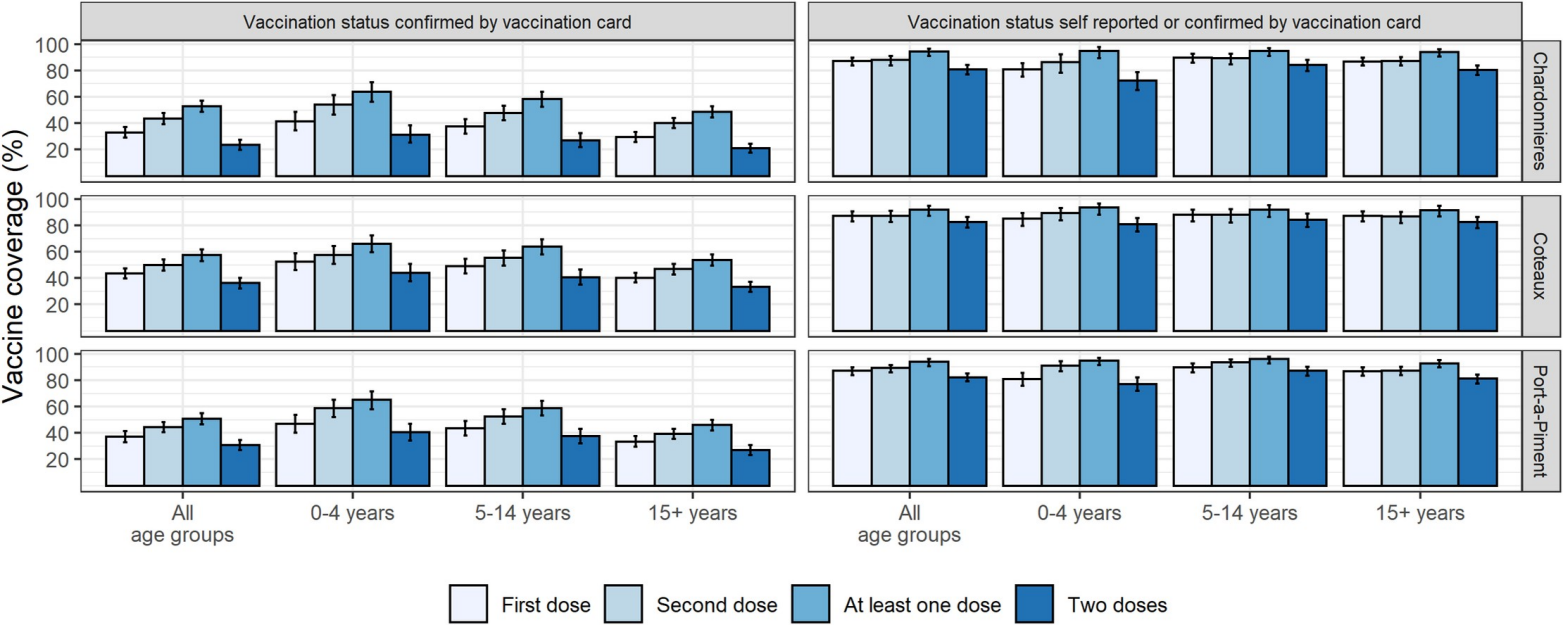
Cholera vaccination coverage by Commune, age group and assessment method, South Department, Haiti, 2017.

**Fig 2:**
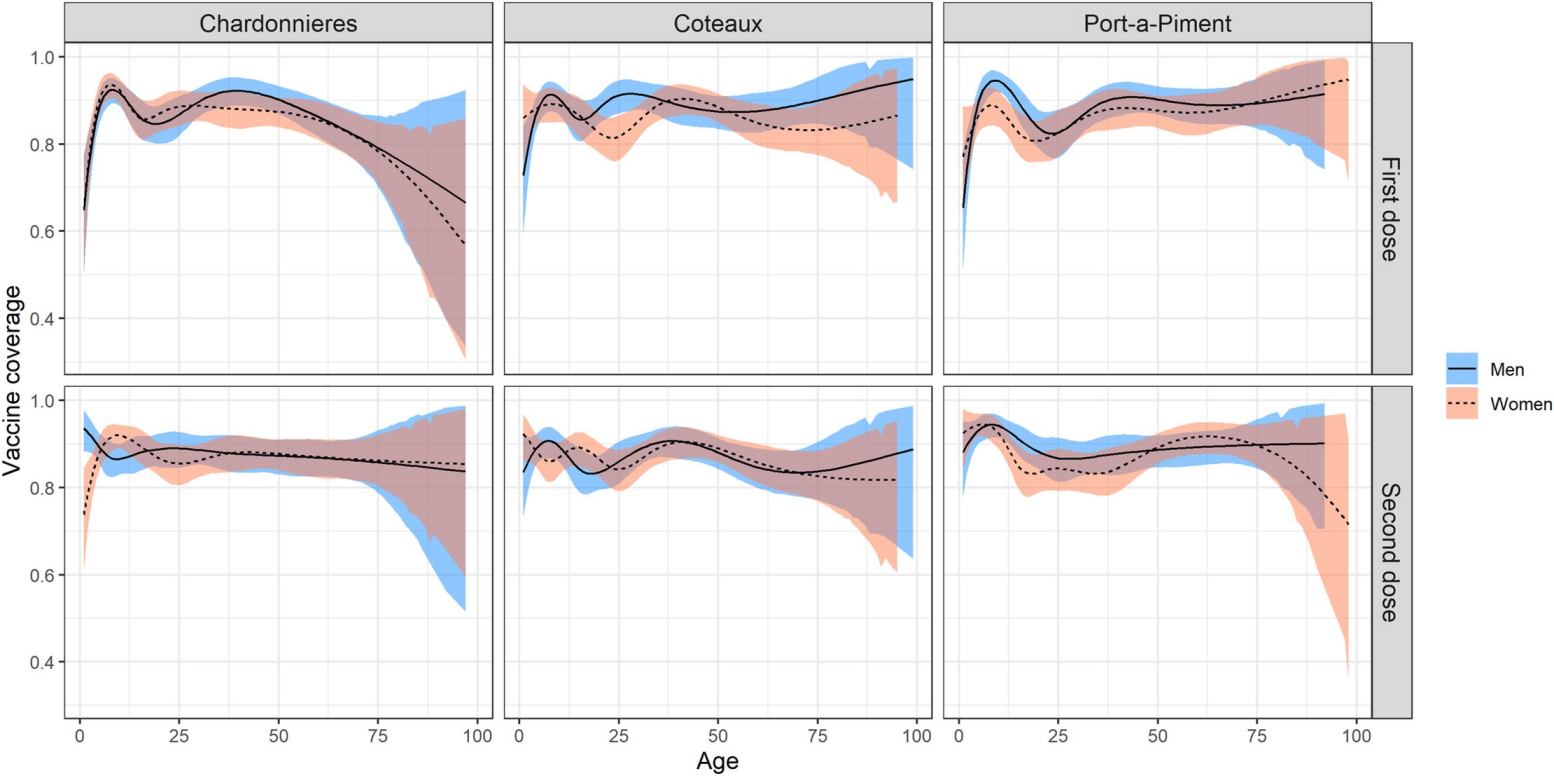
Self-reported cholera vaccination coverage by Commune, age and gender, South Department, Haiti, 2017.

**Fig 3:**
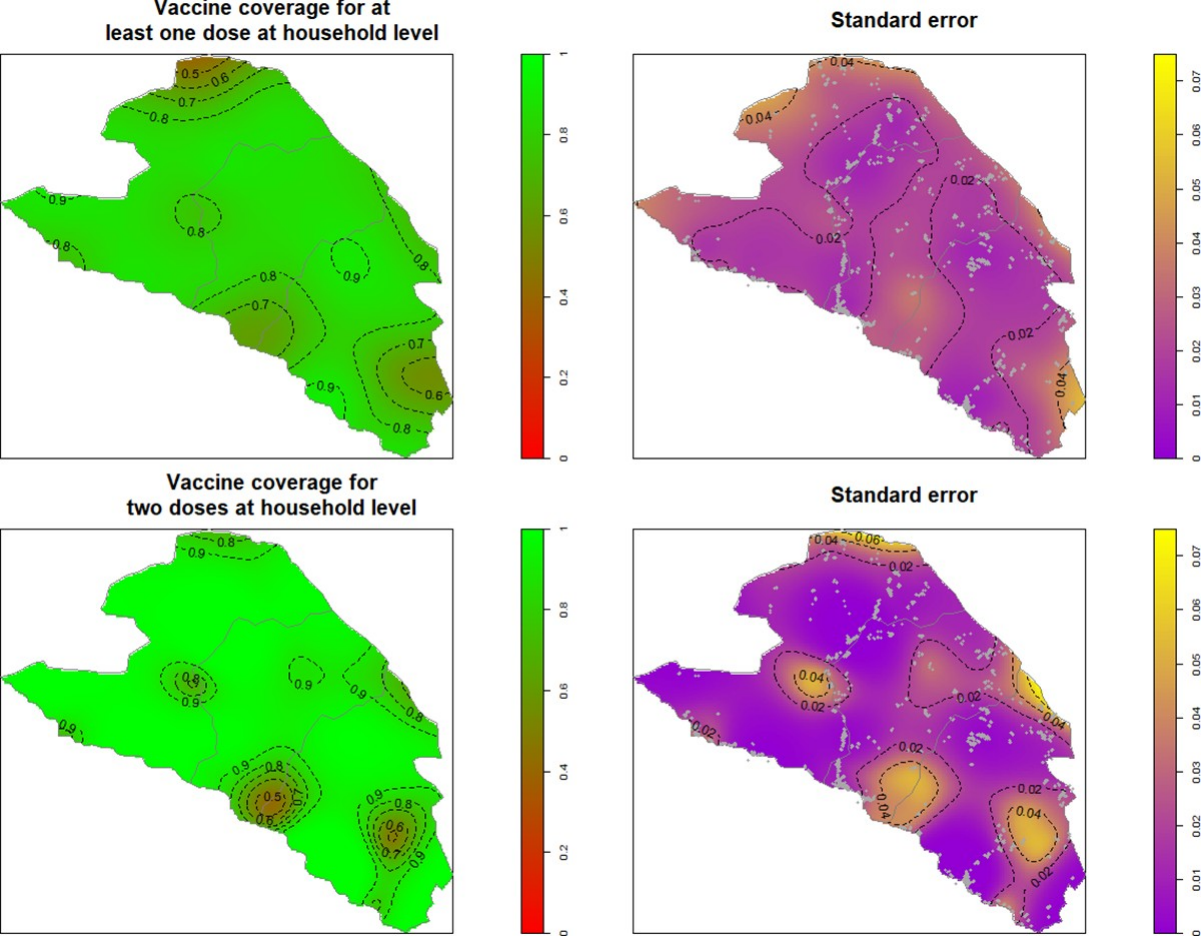
Interpolation of the spatial variation in self-reported cholera vaccination coverage, with standard error and position of GPS points, South Department, Haiti, 2017. Maps produced using R using GADM shapefiles for the boundaries.

Self-reported coverage for at least one dose ranged from 91.8% (87.0–94.9) in Côteaux to 94.2% (90.8–96.4) in Chardonnières (Fig 1). Self-reported coverage for two doses ranged from 80.7% (76.8–84.1) in Chardonnières to 82.6% (78.1–86.4) in Côteaux. Card-confirmed coverage for at least one dose ranged from 50.8% (46.7–54.9) in Port-à-Piment to 57.3% (53.0–61.6) in Côteaux (Fig 1). Card-confirmed coverage for two doses ranged from 23.5% (19.9–27.5) in Chardonnières to 36.1% (32.2–40.1) in Côteaux. Coverage was similar across age groups, with self-reported coverage for at least one dose ranging from 91.5% (86.8–94.7) among 15+ year olds in Côteaux to 96.1% (92.5–98.0) in 5–14 year olds in Port-à-Piment. The drop-out rate was similar in the three communes ranging from 5.3% (3.3–7.3) in Côteaux to 7.4% (4.9–9.8) in Chardonnières. Coverage was similar across both genders (Fig 2). For the first dose, adolescent and young women in Côteaux and Port-à-Piment had a slightly lower coverage than men of the same age. For the second dose, the young girls in Chardonnières had a lower coverage than boys of the same age. There was low uptake in the first dose for very young children of both genders, due to ineligibility. Despite high coverage overall, there was some spatial variation in coverage (Fig 3). Note, the northern zone of Chardonnières was excluded from interpolation because the area is sparsely populated and no households were sampled there.

### Population movements

The vast majority of participants in the three communes were already living there at the time of the first dose campaign: 98.6% (97.6–99.1) at Chardonnières. 99.5% (99.1–99.8%) in Côteaux and 98.7% (98.1–99.1) in Port-à-Piment.

### Preferential vaccine delivery strategy used by the participants

Both door-to-door and fixed-site strategies were widely used. In Côteaux, the majority of participants reported receiving the vaccine from a fixed site: 56.3% (52.5–60.0%) for dose one and 56.4% (52.5–60.1) for dose two. In Chardonnières and Port-à-Piment the majority reported receiving the vaccine door-to-door: 61.3% (57.6–64.9) for dose one and 58.9% (55.0–62.7) for dose two at Chardonnières and 54.7% (51.0–58.3) for dose one and 53.7% (50.0–57.3) for dose two at Port-à-Piment.

### Source of information

Information on the vaccination campaigns was mainly obtained through criers, with 52.6% (49.0–56.2) in Chardonnières, 67.3% (64.0–70.4) in Côteaux and 54.2% (51.0–57.4) in Port-à-Piment hearing about the second-dose campaign this way (Table 1).

**Table 1:** Source of information about the vaccination campaign by dose and Commune, South Department, Haiti, 2017.

	Dose 1						Dose 2					
	Chardonnières (n = 3081)		Côteaux (n = 3109)		Port-à-Piment (n = 2895)		Chardonnières (n = 3081)		Côteaux (n = 3109)		Port-à-Piment (n = 2895)	
	%	95% CI	%	95% CI	%	95% CI	%	95% CI	%	95% CI	%	95% CI
I was not aware that there was a vaccination campaign	0.3	0.1–0.6	0.2	0.1–0.7	0.3	0.1–0.6	0.3	0.1–0.6	0.1	0.0–0.4	0.2	0.0–0.9
Television / Radio	0.1	0.0–0.3	0.1	0.0–0.3	1.9	1.2–3.1	0.1	0.0–0.3	0.1	0.0–0.3	2.1	1.3–3.3
Newspaper	0.1	0.0–0.4	0.1	0.0–0.4	0.6	0.3–0.9	0.7	0.4–1.2	0.5	0.2–1.1	1.3	0.8–2.2
Church / mosque	3.8	2.8–5.0	1.4	0.9–2.2	7.5	5.8–9.5	3.7	2.0.7–5	1.1	0.6–1.8	7.7	6.0–9.7
By friends / neighbours	5.3	4.0–7.0	3.0	2.0–4.5	6.7	5.1–8.6	5.2	3.9–6.9	3.1	2.0–4.6	6.6	5.1–8.5
Village leader / neighbourhood leader / political leader	0.6	0.3–1.2	1.2	0.7–2.1	1.0	0.5–1.8	0.6	0.3–1.1	1.2	0.0.7–2	0.9	0.5–1.7
Vaccinators / nursing staff	14.9	12.4–17.9	11.5	9.4–14.1	8.7	6.9–10.9	15.0	12.4–17.9	11.7	9.5–14.4	8.5	6.7–10.7
Family members	7.8	6.4–9.4	6.3	5.2–7.6	6.6	5.4–8.1	7.7	6.3–9.2	6.3	5.2–7.5	6.5	5.3–8.0
When pre-marking homes	0.3	0.1–0.8	0.2	0.0–0.6	0.2	0.1–0.5	0.2	0.1–0.7	0.1	0.0–0.7	0.2	0.1–0.5
Banners. flyers. or posters	4.9	3.3–7.2	4.2	2.8–6.2	3.8	2.6–5.7	4.8	3.2–7.1	4.1	2.8–6.1	3.8	2.5–5.6
Schools / markets	6.0	4.8–7.6	2.9	2.2–3.7	5.2	4.2–6.4	7.4	6.0–9.0	3.2	2.5–4.1	5.9	4.8–7.1
Criers	53.5	49.8–57	67.5	64.2–70.7	55.2	52.0–58.4	52.6	49.0–56.2	67.3	64–70.4	54.2	51–57.4
Community meetings	0.4	0.2–0.7	0.2	0.1–0.4	0.2	0.0–1.3	0.2	0.1–0.4	0.2	0.0–0.8	0.3	0.1–1.2
An NGO	0	0.0–0.0	0	0.0–0.0	0.3	0.1–0.6	0	0.0–0.0	0	0.0–0.0	0.2	0.1–0.5
Other	2.1	1.3–3.6	1.2	0.8–1.9	1.8	1.2–2.8	1.8	1.0–3.2	1.1	0.7–1.8	1.6	1.0–2.4

### Adverse events

Adverse events were uncommon: for Chardonnières, Côteaux and Port-à-Piment the proportion of those vaccinated that reported adverse events was 4.6% (3.8–5.7), 2.9% (2.3–3.7) and 5.7% (4.6–6.9) following dose one and 4.9% (4–6.1), 3.1% (2.4–3.9) and 6.8% (5.6–8.3) following dose two respectively. Among those who reported adverse events, the most common were weakness/fatigue and headaches. The proportion attending a health centre following the events ranged from 4.8% in Port-à-Piment to 16% in Côteaux (Table 2).

**Table 2:** Adverse events reported by dose and Commune, South Department, Haiti, 2017.

		Dose 1							Dose 2					
		Chardonnières (n = 2849)		Côteaux (n = 2961)			Port-à-Piment (n = 2728)		Chardonnières (n = 2849)		Côteaux (n = 2961)		Port-à-Piment (n = 2728)	
		%	95% CI	%	95% CI	%		95% CI	%	95% CI	%	95% CI	%	95% CI
Rate of adverse events		4.6	3.8–5.7	2.9	2.3–3.7	5.7		4.6–6.9	4.9	4–6.1	3.1	2.4–3.9	6.8	5.6–8.3
Type of adverse event	Nothing	0.8	0.1–5.3	1.1	0.2–8	0.7		0.1–4.6	1.4	0.3–5.6	3.3	1–9.9	0.5	0.1–3.8
	Fever	2.3	0.5–9.4	3.4	1.1–10.5	0.7		0.1–4.7	2.8	0.8–9.1	3.3	1–10	2.2	0.8–5.6
	Diarrhoea	9.8	4.8–19.1	12.6	6.8–22.4	13.2		8.1–20.7	7.1	3.8–12.8	11.0	5.1–22.2	12.9	7.4–21.5
	Abdominal pain	9.1	5–16.1	2.3	0.6–9	9.9		5.7–16.4	7.8	4.2–14.2	4.4	1.6–11.3	8.1	4.8–13.3
	Nausea	7.6	3.9–14.1	5.7	2.4–13	11.2		6.7–18	5.0	2.2–11	3.3	1–9.9	11.3	7.2–17.2
	Vomiting	4.5	2–9.9	4.6	1.4–14.4	5.3		2.4–11	6.4	3.3–11.9	3.3	1–10	5.4	2.5–11.2
	Headache	26.5	19.1–35.5	26.4	17.7–37.5	17.8		11.5–26.5	23.4	16.3–32.4	22.0	14.7–31.6	22.0	16–29.5
	Weakness / fatigue	26.5	19.4–35.1	32.2	23.1–42.8	30.9		23.4–39.7	36.2	28–45.3	39.6	29.8–50.2	25.8	19.6–33.1
	Other	12.9	7.8–20.5	11.5	5.6–22	10.5		5.7–18.7	9.9	5.8–16.6	9.9	4.8–19.4	11.8	7.3–18.6
	Consultation in a health centre following adverse event	13.6	8–22.3	16.1	9.2–26.7	5.9		3.1–11.1	12.8	7.7–20.5	5.5	2.3–12.5	4.8	2.5–9.2

### Reasons for non-vaccination

Among those who did not receive vaccination, the most frequently reported reason was absence/non-availability due to work or illness. In Chardonnières, Côteaux and Port-à-Piment this reason was given by 55.6% (46.3–64.4), 56.3% (46.0–66.2) and 40.9% (32.2–50.2) of non-vaccinees for dose one, and 37.3% (27.4–48.3), 36.8% (25.9–49.3) and 41.5% (31.9–51.8) for dose two.

## Discussion

Vaccination coverage was high, with Chardonnières, Côteaux and Port-à-Piment reporting 80.7%, 82.6% and 82.3% receiving both doses and 94.2%, 91.8% and 93.8% receiving at least one dose respectively. Coverage was similar across each age group and between males and females, though there was some small-area spatial variation. The main reason for non-vaccination was absence due to work or illness. Both door-to-door and fixed-site strategies were widely used to access vaccination and most people heard about the campaign through local criers. Adverse events were uncommon. This was a well-powered study with a robust sampling strategy and high levels of participation.

Adult males had a similar vaccination coverage to females in the same age group, in contrast with previous experience that they are harder to reach because of their occupations [4, 13, 14]. A decline in economic activity following Hurricane Matthew in these agricultural communes could have explained this to some extent and perhaps contributed to high coverage. The six month delay for the second dose would have meant reduced protection, but also offered more time to mobilise the community to promote completion of vaccination. The spatial variation observed in coverage is to be interpreted with caution where populations are very sparsely populated, such as the northern part of Chardonnières. Some spatial variation is difficult to completely avoid, especially in contexts with hard-to-reach areas.

The use of a dual door-to-door and fixed-site strategies varied somewhat by Commune but both were widely utilised. This finding highlights the usefulness of a mixed approach that offers more opportunities to access the vaccine. Door-to-door vaccination may have been particularly important in areas with limited accessibility to healthcare facilities and other fixed vaccination sites. The success of the criers in communicating the vaccination campaigns highlights the importance of community engagement and mobilisation.

The survey coverage estimate was substantially higher than administrative coverage estimates. This was likely due to the limitations of the denominator data, which was not census based but used cluster survey methods, and population movements [12]. The population denominator may have overestimated true population size post-hurricane. This highlights the limitations of using such administrative data to evaluate vaccination coverage and the need for up-to-date population denominators and specific vaccination coverage surveys where necessary. Most study participants were already resident at the time of the first dose campaign suggesting there was no large inward population movement between the first dose and the time of the survey.

There is growing experience of using mass vaccination in cholera outbreaks, enabled by the creation of the global stockpile. Where there are shortages, modelling suggests even a single dose has demonstrable efficacy and may be important in outbreak control [15]. This high-coverage two-dose campaign likely contributed to preventing cholera cases in the aftermath of Hurricane Matthew, [16], however protection is unlikely to last beyond three years [17] and the effect of a six-month delay between doses is not known. Vaccine effectiveness remains to be estimated, and further studies will be important to fully evaluate this intervention. There was a low rate of adverse events as has been seen with Shancol [18, 19].

### Limitations

The main limitation of this study is the self-reported vaccination status. The card-confirmed vaccination status is also reported but this likely underestimates true coverage because cards are often misplaced. Relying on self-reported status could lead to an overestimation of coverage, as people may prefer to report that they have vaccinated their families when asked. On the other hand, relying on self-report could lead to an underestimation, as people may have forgotten about the vaccination, or confused it with other vaccination campaigns. Self-report for cholera vaccine is probably more reliable than for other vaccines as it is administered orally, unlike most other vaccines. In this case, it was also perhaps more memorable for having been offered in exceptional circumstances during an outbreak alongside a high-profile campaign [20]. Another limitation is that only the head of the household responded to the questionnaire on behalf of their family and other household members may not have been present during the visit to confirm the information. It is not clear how this limitation would have affected coverage estimates. Finally, no information could be collected on households that had completely left the area, since at least one member needed to be present to answer the questions, but again we do not know whether those families that left are more or less likely to have been vaccinated.

### Conclusion

High vaccination coverage was achieved in this campaign. The use of a dual strategy to deliver the vaccines and extensive community mobilisation made it possible to achieve a high coverage in this rural setting with limited accessibility. This experience supports the use of mass vaccination during outbreaks in similar settings utilising multiple delivery strategies and community engagement as a feasible control measure. The addition of Euvichol to the stockpile should help alleviate shortages and extend the range of situations where vaccination can be considered as an intervention. Further studies are needed to assess the effectiveness of Euvichol in outbreak control. Where administrative data is limited or where population data is unreliable, cluster surveys provide an effective method to assess vaccination coverage.
